# Clinical features of irreversible rejection after allogeneic uterus transplantation in cynomolgus macaques

**DOI:** 10.1038/s41598-020-70914-1

**Published:** 2020-08-17

**Authors:** Iori Kisu, Katsura Emoto, Yohei Masugi, Yohei Yamada, Kentaro Matsubara, Hideaki Obara, Yusuke Matoba, Kouji Banno, Yojiro Kato, Yoko Saiki, Iori Itagaki, Ikuo Kawamoto, Chizuru Iwatani, Mitsuru Murase, Takahiro Nakagawa, Hideaki Tsuchiya, Hirohito Ishigaki, Hiroyuki Urano, Masatsugu Ema, Kazumasa Ogasawara, Daisuke Aoki, Kenshi Nakagawa, Takashi Shiina

**Affiliations:** 1 Department of Obstetrics and Gynecology, Keio University School of Medicine, 35 Shinanomachi , Shinjuku-ku, Tokyo 1608582 Japan; 2 Department of Pathology, Keio University School of Medicine, Shinjuku, Tokyo 1608582 Japan; 3 Department of Pediatric Surgery, Keio University School of Medicine, Shinjuku, Tokyo 1608582 Japan; 4 Department of Surgery, Keio University School of Medicine, Shinjuku, Tokyo 1608582 Japan; 5 Department of Surgery, Division of Gastroenterological and General Surgery, School of Medicine, Showa University, Shinagawa, Tokyo 1428666 Japan; 6 Department of Anesthesiology, Saiseikai Kanagawaken Hospital, Yokohama, Kanagawa 2210821 Japan; 7 Research Center for Animal Life Science, Shiga University of Medical Science, Ōtsu, Shiga 5202192 Japan; 8 The Corporation for Production and Research of Laboratory Primates, Tsukuba, Ibaraki 3050003 Japan; 9 Department of Pathology, Shiga University of Medical Science, Ōtsu, Shiga 5202192 Japan; 10 Safety Research Center, Ina Research Inc., Ina, Nagano 3994501 Japan; 11 Department of Molecular Life Science, Division of Basic Medical Science and Molecular Medicine, Tokai University School of Medicine, Hiratsuka, Kanagawa 2591193 Japan

**Keywords:** Experimental models of disease, Reproductive disorders, Infertility

## Abstract

Uterus transplantation (UTx) is a potential option for women with uterine factor infertility to have a child. The clinical features indicating irreversible rejection of the uterus are unknown. In our experimental series of allogeneic UTx in cynomolgus macaques, six female macaques were retrospectively examined, which were unresponsive to treatment with immunosuppressants (i.e. irreversible rejection). Clinical features including general condition, hematology, uterine size, indocyanine green (ICG) fluorescence imaging by laparotomy, and histopathological findings of the removed uterus were evaluated. In all cases, general condition was good at the time of diagnosis of irreversible rejection and thereafter. Laboratory evaluation showed temporary increases in white blood cells, lactate dehydrogenase and C-reactive protein, then these levels tended to decrease gradually. In transabdominal ultrasonography, the uterus showed time-dependent shrinkage after transient swelling at the time of diagnosis of irreversible rejection. In laparotomy, a whitish transplanted uterus was observed and enhancement of the transplanted uterus was absent in ICG fluorescence imaging. Histopathological findings in each removed uterus showed hyalinized fibrosis, endometrial deficit, lymphocytic infiltration and vasculitis. These findings suggest that uterine transplantation rejection is not fatal, in contrast to rejection of life-supporting organs. Since the transplanted uterus with irreversible rejection atrophies naturally, hysterectomy may be unnecessary.

## Introduction

Uterus transplantation (UTx) has become a potential option for women with uterine factor infertility to have a child, following the first successful delivery by Brännström et al. in Sweden in 2014^1^. This major achievement attracted considerable attention worldwide, and many centers have recently begun clinical application of UTx, leading to more than 60 procedures and 18 live births^2^. Despite this success, UTx is still in an experimental stage and there are many clinical and technical issues to be resolved for its full clinical establishment^3^. In particular, rejection remains as a concern in this procedure. There have been several reported cases of rejection after UTx in humans^4–9^ and pathological criteria for uterine rejection have been proposed by the group in Sweden^10^. All of these cases overcame rejection with subsequent immunosuppressive therapy and some achieved successful live birth after treatment of the rejection^1,8^.

Organ elimination due to rejection after transplantation of a life-supporting organ generally results in a fatal outcome. However, UTx is not a vital organ transplantation, and organ elimination due to rejection might not lead to life-threatening conditions. However, there are no reports of a uterus that became non-functional or was removed due to irreversible rejection that was resistant to treatment, and the clinical features indicating irreversible rejection of the uterus are unclear.

Our team in Japan launched UTx research in 2009 using approximately 100 cynomolgus macaques, which are anatomically and physiologically similar to humans. Since then, we have accumulated a large archive of results, including examination of uterine blood flow, surgical procedures for autologous and allogeneic UTx, organ perfusion methods in deceased donor models, immunological response and rejection, and ischemia/reperfusion injury^11^. In this experimental series, we had cases of hysterectomy after allogeneic UTx due to diagnosis of irreversible rejection that was resistant to immunosuppressants.

These cases arose from difficulty and limitations in postoperative managements and monitoring in cynomolgus macaques^12,13^, which are likely to develop rejection and have high antigenicity, in contrast to humans^14^. These events may be of value in understanding rejection in UTx because similar events have not been observed in humans. Therefore, in this study, we retrospectively examined the clinical features associated with irreversible rejection after allogeneic UTx in cynomolgus macaques.

## Results

### Irreversible rejection

In this study, irreversible rejection was defined as progressive rejection in spite of the following treatments. Steroid pulse therapy was administered in all cases and ATG in cases 4 and 5, but the rejections were unresponsive and not overcome by these treatments. Moreover, necrotic change in uterine tissues was often observed histologically. The cases were diagnosed as irreversible rejection on postoperative day (POD) 11, 35, 35, 41, 67 and 206 (Table 1).

**Table 1:** Summary of the timing of irreversible rejection in cases 1–6.

Case	POD of irreversible rejection	Treatment for rejection	POD of autopsy
1	11	Steroid pulse	85
2	35	Steroid pulse	196
3	35	Steroid pulse	126
4	41	Steroid pulse + ATG	104
5	67	Steroid pulse + ATG	103
6	206	Steroid pulse	265

*POD* postoperative day, *ATG* antithymocyte globulin.

### Well-being of the animals

In all cases, general condition of the animals (activity, appetite, bowel movement, vomiting, urination) was good at the time of diagnosis of irreversible rejection and thereafter. No animals did not show body weight loss greater than 15%. In cases 2, 5 and 6, continuous vaginal bleeding was observed before and/or after the day of irreversible rejection.

### Laboratory evaluation

**Figure 1:**
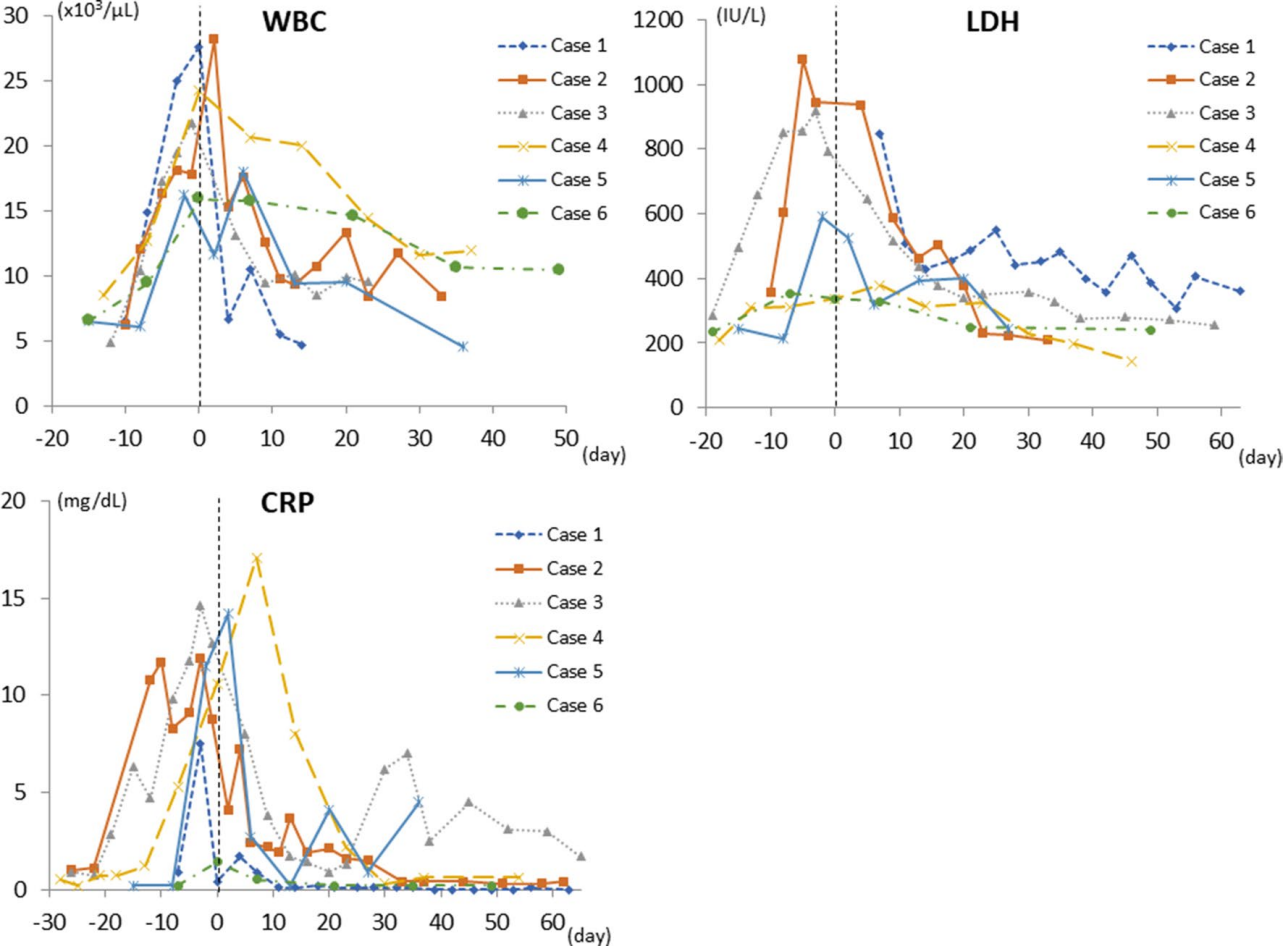
Changes in WBC, LDH and CRP before and after irreversible rejection. WBC, LDH and CRP temporarily increased before and after diagnosis of irreversible rejection, and then tended to decrease gradually (day 0 = day of diagnosis of irreversible rejection).

Hematology and blood chemistry in all animals showed temporary increases in white blood cells (WBC), lactate dehydrogenase (LDH), and C-reactive protein (CRP) before and after rejection was diagnosed (Fig. 1). Thereafter, these levels tended to decrease gradually. There were no marked changes in electrolytes or in liver and renal functions.

### Assessment of the transplanted uterus by ultrasonography

**Figure 2:**
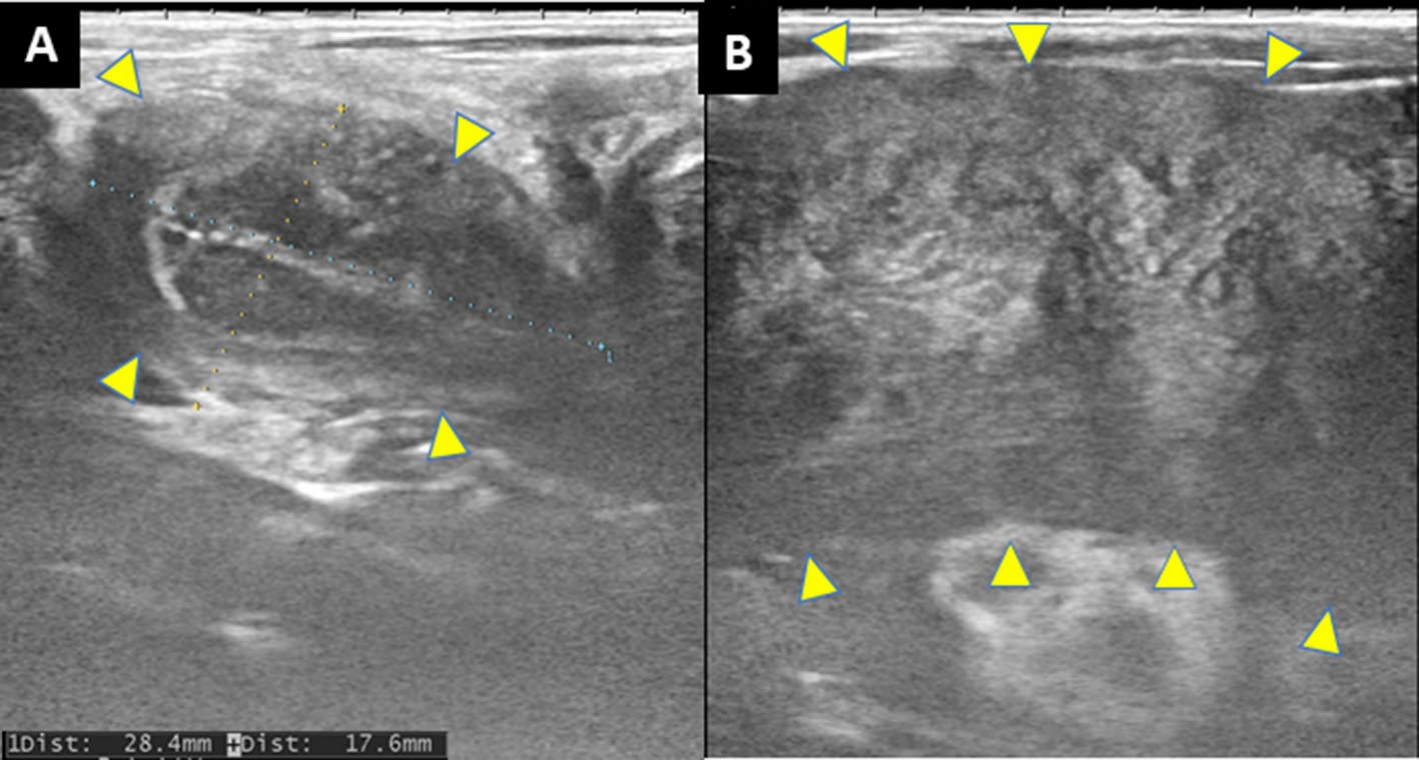
Transabdominal ultrasonography of the long axis of the uterine body in case 5. (**A**) The uterine body (28.4 × 17.6 mm: long axis × anteroposterior diameter) after surgery. (**B**) An enlarged uterine body (60.0 × 28.1 mm: long axis × anteroposterior diameter) without an endometrium was found at the time of diagnosis of irreversible rejection.

**Figure 3:**
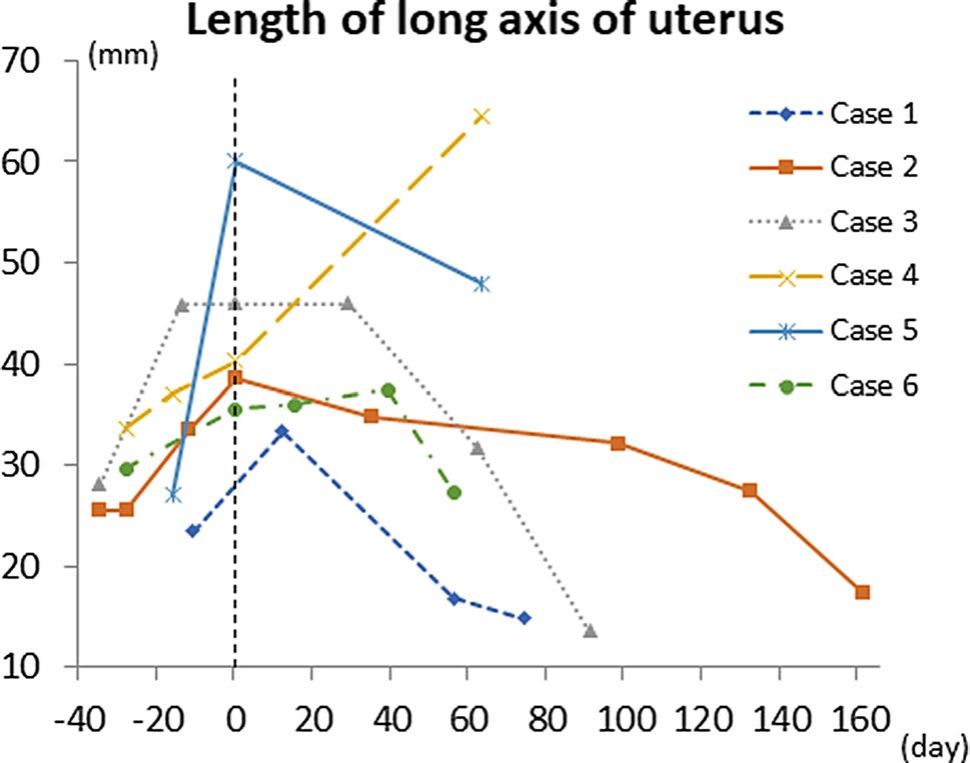
Changes in the long axis diameter of the uterus before and after irreversible rejection. The uterus was swollen at the time of diagnosis of irreversible rejection, and then showed time-dependent shrinkage (day 0 = day of diagnosis of irreversible rejection).

In transabdominal ultrasonography, the uterus was swollen at the time of diagnosis of irreversible rejection (Fig. 2). The uterus then showed time-dependent shrinkage (Fig. 3), which made it difficult to identify blood flow in the uterine artery on duplex Doppler ultrasonography. The endometrium of the uterus was not detected in all cases.

### Gross findings and ICG fluorescence imaging in laparotomy

**Figure 4:**
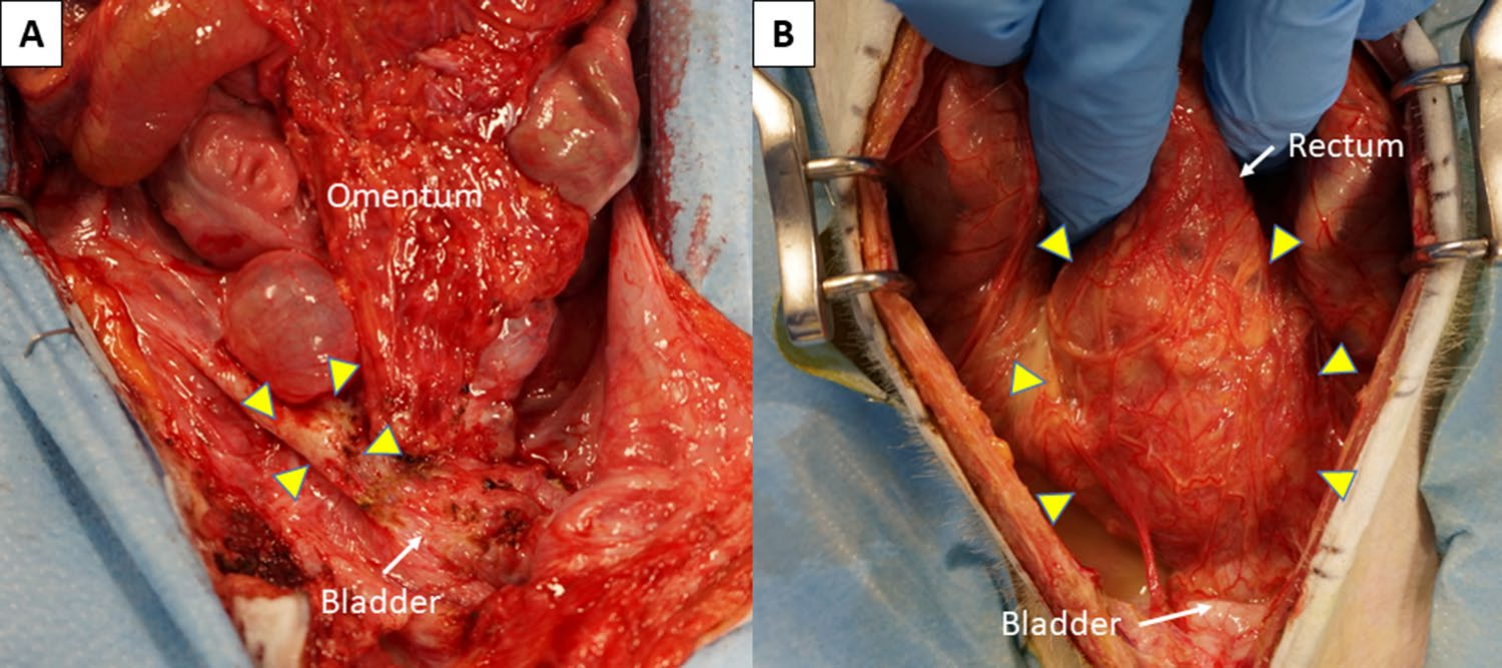
Macroscopic findings in the pelvis at autopsy in case 3 (**A**) and case 4 (**B**). (**A**) A whitish atrophic uterus (yellow triangles) adhered to the omentum and bladder. (**B**) A swollen uterus that was highly adhesive with surrounding tissues, especially to the omentum and rectum.

**Figure 5:**
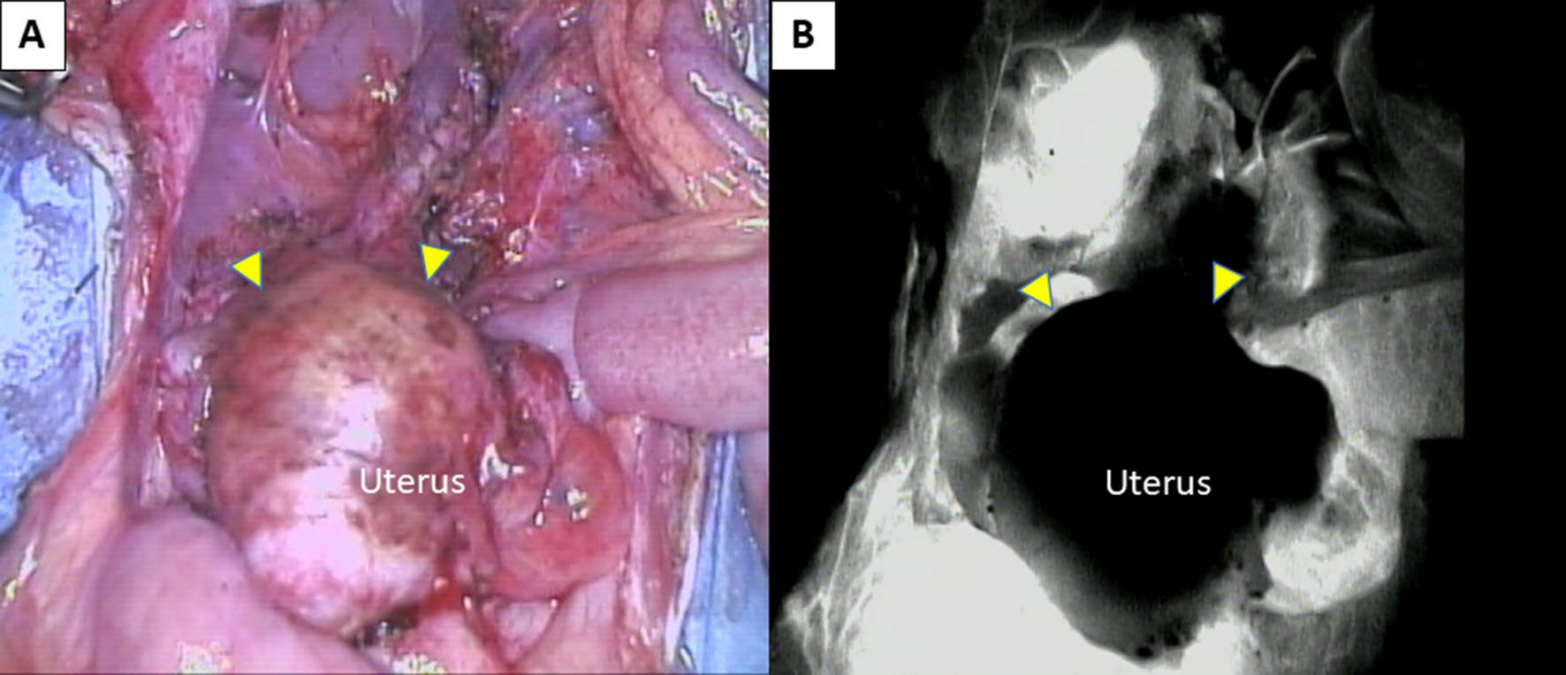
ICG fluorescence imaging of the transplanted uterus at autopsy in case 4. (**A**) A markedly swollen uterus (yellow triangles). (**B**) Enhancement of the grafted uterus (yellow triangles) was absent in ICG fluorescence imaging.

Autopsy in cases 1–6 was performed on POD 85, 196, 126, 104, 103 and 265, respectively, because of irreversible rejection that was resistant to additional immunosuppressant therapy. Laparotomy showed a white shrinking uterus that was highly adhesive with surrounding tissues, especially to the bladder, the greater omentum and rectum (Fig. 4A), in cases other than cases 4 and 5. In cases 4 and 5, a whitish swollen transplanted uterus was observed (Fig. 4B). Moreover, the uteri of all cases were hard and sclerotic by gross diagnosis. In ICG fluorescence imaging, enhancement of the transplanted uterus was absent in all cases (Fig. 5).

### Pathological findings of the removed uterus

**Figure 6:**
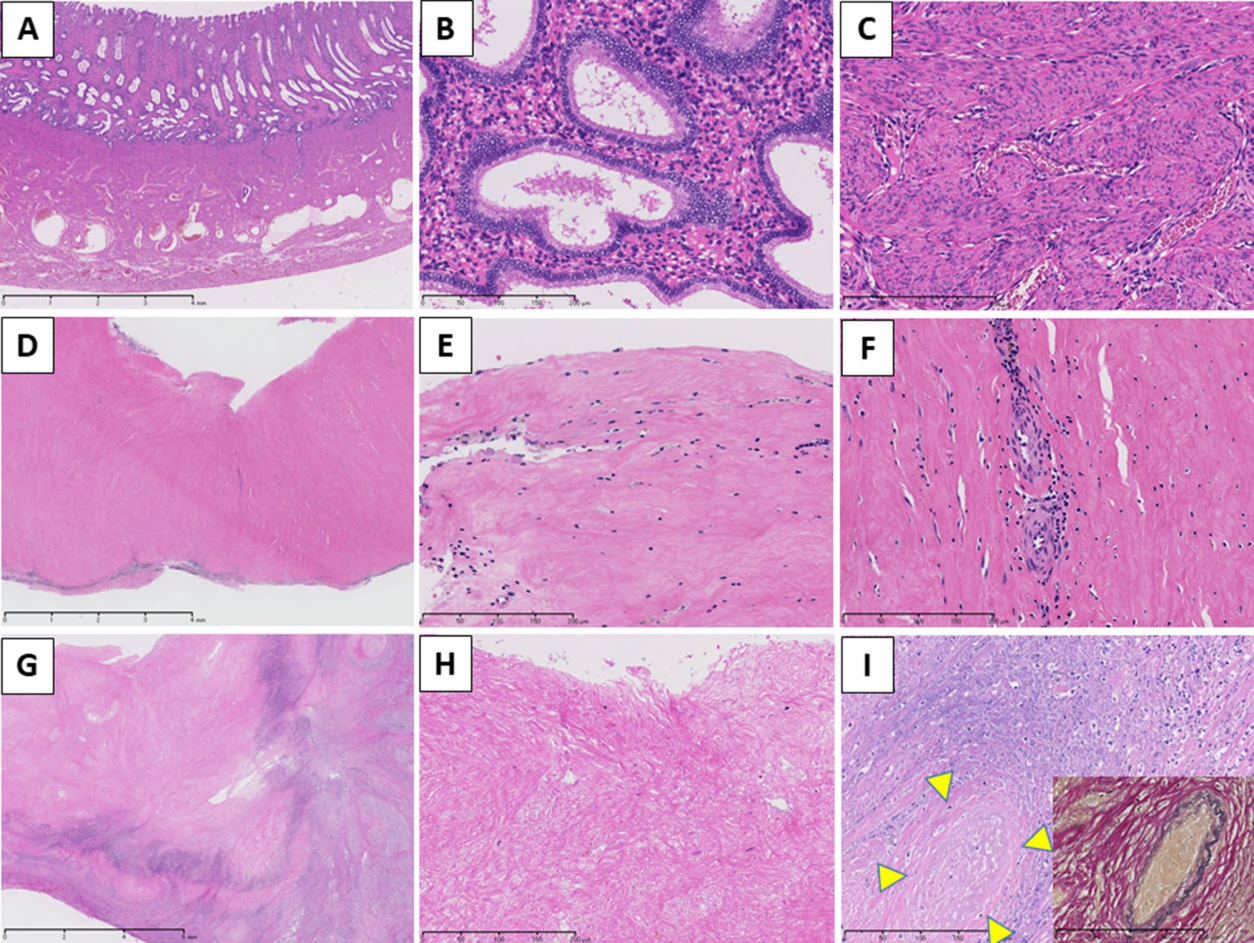
Histopathological findings of normal uterus in a cynomolgus macaque (**A**–**C**) and the removed uterus at autopsy in case 2 (**D**–**F**) and case 4 (**G**–**I**). (**A**) Normal uterine corpus is composed of endometrium, myometrium and perimetrium. (**B**) High power field of endometrium. (**C**) High power field of myometrium. (**D**) The uterine corpus of case 2 demonstrated fibrous change of the whole wall and inflammation in the perimetrium. (**E**) Endometrium was not seen and replaced by sclerotic fibrosis. (**F**) No smooth muscle cells were observed in the myometrium which was also replaced by sclerotic fibrosis. Mild perivascular inflammation was observed. (**G**) The uterine corpus of case 4 demonstrated necrotic change of the whole wall and inflammation in the perimetrium. (**H**) No endometrium but degenerated fibrotic tissue was seen. (**I**) Myometrium showed coagulative necrosis and vascular occlusion (yellow triangles) which was highlighted by Elastica van Gieason stain in the inset. H&E stain (**A**–**I**). Bar = 4 mm (**A**, **D**), 200 µm (**B**, **C**, **E**, **F**, **H**, **I**), and 6 mm (**G**).

Histologically, compared to the normal uterus (Fig. 6A–C), uteri of the six cases showed no endometrium and myometrium which were replaced by sclerotic fibrosis (Fig. 6D–F) or coagulative necrosis (Fig. 6G–I), which suggested of end stage of the rejection. All cases demonstrated perivascular lymphocytic inflammation, and some cases showed vascular occlusion and/or fibrin thrombi. Inflammation was more severe in perimetrium than in endometrium and myometrium. These histologic findings, loss of endometrial and myometrial structure, indicated that these uteri were no longer functional.

## Discussion

An important goal of UTx is improving quality of life (QOL) for women with uterine factor infertility by allowing these women to have a child, in contrast to transplantation of life-supporting organs. UTx has provided great hope to couples with no children due to uterine factor infertility. However, various risks are involved in organ transplantation, and rejection is a particular concern. The antigenicity of a transplanted organ depends on the organ type and is unclear for the uterus. A pregnant uterus allows a non-self organism to develop within, and thus may be presumed to maintain immune tolerance. On the other hand, organs such as skin, small intestine and lungs that are in contact with the external environment have well-developed immune mechanisms that are likely to cause rejection. The uterus is similarly in contact with the external environment via the vagina, and this may explain the strong potential for rejection of a transplanted uterus.

Some cases of human UTx have resulted in uterine rejection^4–9^, but none have required hysterectomy due to refractory rejection. Organ elimination due to rejection after transplantation of a life-supporting organ generally results in a fatal outcome. Since UTx is not life-supporting, but focused on improving QOL, particular attention should be paid to the safety of the healthy recipient. The clinical features of irreversible rejection after UTx are unknown, including the potential mortality that occurs in other organ transplantations, and preparation for unexpected events after UTx is important. Thus, in this study we examined six cynomolgus macaques in which irreversible rejection occurred in our experimental series of allogeneic UTx to clarify the clinical features of irreversible rejection. The main features found were temporary increases in WBC, LDH and CRP before and after irreversible rejection, a swollen uterus at the time of irreversible rejection that then shrinks over time, and a good general condition. In laparotomy, the uterus without uterine blood flow was highly adhesive with surrounding tissues. Pathological findings of the uterus showed hyalinization in the interstitium in all uterine layers, with an endometrial deficit.

The increases in WBC and CRP imply an inflammatory response, and elevated LDH suggests cell breakdown due to rejection. In conventional life-supporting organ transplants, clinical symptoms and biochemical data can be used for monitoring of rejection, instead of biopsy. In contrast, the uterus is not a life-supporting organ and rejection is not immediately life-threatening and is frequently asymptomatic; therefore, no clear findings showing rejection have not been reported in human UTx. The biochemical findings above may be one indicator; however, these findings are often caused by irreversible rejection and it is likely to be difficult to recover uterine function once they occur. Thus, monitoring of rejection by an alternative means to biopsy is more difficult in UTx than in life-supporting organ transplantation.

The swelling of the uterus at the time of rejection shows inflammatory edema due to rejection, and the subsequent shrinkage reflects cell damage, necrotic cell death, and hyalinization and formation of granulation tissues due to ischemia over time, resulting in atrophy. The uteri in cases 4 and 5 were still swollen at autopsy, but it is likely that these uteri will be atrophic thereafter because these cases could not overcome uterine rejection. These processes also occur in life-supporting organs, such as in kidney and liver transplantation.

A major concern in UTx is whether a uterus with irreversible rejection should be removed. Such a uterus is not likely to cause serious problems after rejection and subsequent uterine atrophy and loss of function because the uterus is not a vital organ. This study also showed that general condition remained good in the macaques in which irreversible rejection occurred. Likewise, a good general condition is maintained in macaques with an atrophied uterus after warm ischemia for 8 h, which we used to examine the allowable warm ischemic time and ischemic reperfusion injury of the uterus in cynomolgus macaques^15^. Thus, vital organs result in life-threatening conditions if their function is lost, but the uterus is not critical for survival. This difference from other life-supporting organs is an advantage of non-life-supporting organ transplantation.

In kidney transplantation, chronic allograft dysfunction is a chronic, progressive, and irreversible state of a transplanted kidney and one of the leading causes of allograft loss among kidney transplant recipients. In such a case, hemodialysis is introduced as an alternative life-saving treatment. The graft will gradually be atrophied if it is left in the abdomen, and whether an asymptomatic failed renal allograft should be removed before retransplantation is still controversial. The reported rate of surgical allograft nephrectomy after graft failure varies from 20 to 80%, and mainly depends on the center policy^16^. Some investigators have advocated against removal of an asymptomatic failed allograft due to the morbidity and mortality associated with transplant nephrectomy^17,18^. In contrast, others have contended that a failed allograft is a source of sepsis or chronic inflammation that may lead to complications, and therefore, should be routinely removed. There is no consensus on the timing and indications for allograft nephrectomy.

Unsurprisingly, continuation of immunosuppression after graft failure results in an increase in infectious complications. Therefore, after early graft failure the graft is often removed to prevent acute rejection and allow rapid reduction or complete withdrawal of immunosuppressive medication. In auxiliary partial orthotopic liver transplantation (APOLT), a partial liver graft is implanted in an orthotopic position after leaving behind part of the native liver, which has a potential advantage of immunosuppression withdrawal in acute liver failure^19^. In this technique, withdrawal of immunosuppressants can be gradual, so that the graft undergoes slow rejection and ultimately becomes atrophic and fibrotic, while the native liver continues to regenerate to compensate for the loss in graft volume. However, the atrophic graft may have to be removed after immunosuppression withdrawal if it becomes infected^20^.

Given this background, it is uncertain if an atrophic uterus after irreversible rejection should be left in the pelvis or removed. Continuous rejection under immunosuppressants leads to promote infection because the uterine cavity is in contact with the outside of the body through the vagina, whereas kidney and liver do not contact to the outside directly. In the first Swedish trial of UTx, the patient had repeated intrauterine infection after UTx and the uterus had to be resected^5^. We also experienced this situation in cynomolgus macaques that had abscesses temporarily present in the uterine cavity after allogeneic UTx in which continuous rejection occurred^14^. Since organ transplant recipients receive immunosuppressants, they have higher risks for infection than general patients. Intraabdominal infection is occasionally fatal and hysterectomy should be the best choice to prioritize the safety of the recipient in such a situation.

One concern with hysterectomy is that the rejected uterus is likely to be strongly adhered to the surrounding tissues due to inflammation, which leads to cause more operative complications than in a regular hysterectomy. Furthermore, unless uterine infection occurs, the transplanted graft will atrophy naturally. This natural course supports the opinion that a reoperation for resection of the graft is unnecessary and is likely to lead to risks and burdens for the patient. In reports in humans, post-delivery operations have been routinely performed to allow withdrawal of immunosuppressants to decrease the risk of infection. The question of whether to perform hysterectomy for irreversible uterine rejection, an atrophied uterus, or a transplanted uterus after delivery clearly requires further discussion. Moreover, this study has a limitation that these results in cynomolgus macaques cannot always be extrapolated to humans even if nonhuman primate models have the anatomic and physiologic similarities of their reproductive organs and immune systems to humans.

In conclusion, irreversible rejection after allogeneic UTx in cynomolgus monkeys was associated with increases in WBC, LDH and CRP and uterine shrinkage after transient swelling. General condition was good, even after the uterus failed due to rejection, which suggests that uterine transplantation rejection is not fatal, in contrast to rejection of life-supporting organs.

## Materials and methods

### Animals

In our experimental series of allogeneic UTx in cynomolgus macaques, six female recipients of macaques (cases 1–6) (Macaca fascicularis, age 6–13 years; average body weight, 3.85 ± 0.91 kg [mean ± standard deviation]) were retrospectively examined. The six recipients and their donors had compatible ABO blood type and a high degree of polymorphism in the major histocompatibility complex (MHC) gene. All of the recipients were diagnosed with uterine rejection after allogeneic UTx and were unresponsive to treatment with immunosuppressants (i.e. irreversible rejection). The study was performed in accordance with the recommendations in the Guide for the Care and Use of Laboratory Animals of the National Research Council, and was approved by the Animal Care and Use Committee of the Research Center for Animal Life Science, Shiga University of Medical Science, Japan (permit numbers: 2013-4-2, 2016-4-8 and 2019-3-12).

### Immunosuppressive treatment

The animals received immunosuppressive treatment as shown in Table 2. Immunosuppressive protocol varied in these animals. As induction treatment, animals received antithymocyte globulin (ATG) (10 mg/kg; Thymoglobulin, Genzyme, Cambridge, MA, USA) intravenously on postoperative day (POD) 0 (the day of surgery) in cases 2 and 3, and 20 mg/kg of ATG on POD 0 and POD 2 in cases 4–6. Rituximab (2 mg/kg; Rituxan; Genentech, San Francisco, CA, USA) before approximately 3 weeks (within 17–23 days before the surgery) and on POD 0 was also given intravenously on POD 0 and POD 2 in case 5 and 6. Maintenance treatment consisted of tacrolimus (TAC) (Prograf; Astellas Pharma) given orally twice a day in case 1, cyclosporine (CyA) (Sandimmune:Novartis, Basel, Switzerland) given subcutaneously in cases 2 and 3, and TAC (Prograf;Astellas Pharma, Tokyo, Japan) given intramuscularly in cases 4–6. The target trough levels for CyA and TAC up to 1 month after surgery were planned to be in the ranges of 300–400 ng/mL and 15–20 ng/mL, respectively, and thereafter were adjusted as required. As another maintenance treatment,mycophenolate mofetil (MMF) (40–100 mg/kg; Cellcept; Chugai Pharmaceutical, Tokyo, Japan) was administered orally from recovery of appetite after surgery in case 2, 4, 5 and 6. Methylprednisolone (10 mg/kg; Solu-Medrol; Pfizer, NY, USA) was injected intravenously on POD 0 and then injected intramuscularly daily starting on POD 1. The dose of methylprednisolone was gradually tapered. If rejection occurred, steroid pulse therapy of 10 mg/kg methylprednisolone for 2 days was administered and gradually tapered. When the rejection was refractory to conventional steroid pulse treatment, antithymocyte globulin (ATG) were added.

**Table 2:** Immunosuppressive treatment in cases 1–6.

Case	Induction treatment	Maintenance treatment
1	None	Tac + mPSL
2	ATG	CyA + MMF + mPSL
3	ATG	CyA + mPSL
4	ATG	Tac + MMF + mPSL
5	Rxm + ATG	Tac + MMF + mPSL
6	Rxm + ATG	Tac + MMF + mPSL

*ATG* antithymocyte globulin, *CyA* cyclosporine, *MMF* mycophenolate mofetil, *mPSL* methylprednisolone, *Tac* tacrolimus, *Rxm* rituximab.

### Postoperative observation

After allogeneic UTx, these cynomolgus macaques were reared in each cage under the following environmental conditions: temperature of 23–29 °C, humidity of 35–75%, lighting of 12 h/day, and toys to play with all day long. The general condition of the animals (activity, appetite, bowel movement, vomiting, urination) and the laparotomy wound were evaluated daily. Laboratory assessments including hematology, blood chemistry and trough levels of CyA and TAC were performed three times per week for the first two postoperative weeks, twice per week for 2 months, and weekly thereafter. To monitor for potential rejection, transabdominal ultrasonography and transvaginal biopsy of the transplanted uterine tissues of uterine cervix and body by using clamps (Storz 5Fr; Karl Storz) in case 1 and a puncture needle (BARD MAX-CORE; BD [C.R. Bard, Inc.], Tempe, AZ) in case 2–6 were routinely conducted under anesthesia monthly or when considered necessary (whenever graft rejection was suspected). The animals were fed a commercial monkey diet once daily, with supplemental fruits and vegetables seven times weekly before and after the procedure. Lactated Ringer’s solution was administered i.v. if there was a change in the post-operative condition, such as appetite loss or dehydration. Antibiotics and sufficient painkiller were also administered after the invasive procedure. Animals were euthanized if severe weakness, weight loss or abnormal behavior was seen after the procedure or when determined to be necessary by veterinary staff and investigators.

### Laparotomy and indocyanine green (ICG) fluorescence imaging

When irreversible rejection was diagnosed, laparotomy was performed and ICG fluorescence imaging of the uterus was conducted. ICG (Diagnogreen 0.5%; Daiichi Pharmaceutical, Tokyo, Japan) was injected intravenously and the blood supply to the uterus was monitored in real time^21^. An increasing intensity of fluorescence was displayed using the Photodynamic Eye Neo system (Hamamatsu Photonics K.K., Hamamatsu, Japan). Grafted uteri were then removed and evaluated pathologically.

### Endpoints

Clinical features including general condition, body weight loss, hematology, uterine size by transabdominal ultrasonography, intraabdominal gross findings and ICG fluorescence imaging by laparotomy, and histopathological findings of the removed uterus were evaluated. Day 0 was defined as the day when rejection was diagnosed as irreversible based on the course and pathological findings of biopsy.
